# A Multi-Indicator Fusion-Based Technique for the Identification of Acoustic Emission Signals During Rock Failure

**DOI:** 10.3390/s26092759

**Published:** 2026-04-29

**Authors:** Dexian Li, Hongwei Wang, Dengyu Wang, Xuemei Wang, Lianhui Li, Zhongwei Pei, Ying Wang

**Affiliations:** 1 National Key Laboratory of Nickel and Cobalt Associated Minerals Resources Development and Comprehensive Utilization, Jinchang 737100, China; 255502046@csu.edu.cn (D.L.); 18189459062@163.com (D.W.); lianhuili1234@163.com (L.L.); 15197401779@163.com (Y.W.); 2 School of Resources and Safety Engineering, Central South University, Changsha 410083, China; hongwei.wang@csu.edu.cn (H.W.); 245512113@csu.edu.cn (X.W.)

**Keywords:** acoustic emission, waveform segmentation, arrival-time correction

## Abstract

**Highlights:**

**What are the main findings?**

- An energy-envelope-based segmentation strategy is developed to separate dense and overlapping AE waveforms without relying on fixed timing parameters.
- A multi-indicator template sliding-window identification scheme (correlation, ring count, rise time, and energy) is proposed to detect rock-failure-related AE events and refine arrival picking via time-difference correction.

**What are the implications of the main findings?**

- The proposed workflow improves AE event detection and picking reliability under strong attenuation, non-stationarity, and missing-channel recordings, enabling more accurate localization in rock failure tests.
- The method provides a practical foundation for real-time damage monitoring and early-warning of rock instability in geotechnical engineering applications.

**Abstract:**

With the widespread application of acoustic emission (AE) technology in geotechnical engineering, effectively separating and identifying dense AE signals generated during rock fracturing remains a critical challenge. This study proposes an AE event identification technique based on waveform energy envelopes and multi-indicator characteristic parameters. First, the waveform energy envelope is used to adaptively segment dense and partially overlapping AE waveforms without relying on fixed timing parameters. Then, a template sliding-window scan integrating waveform correlation, ring count, rise time, and signal energy is performed to identify candidate AE events. In addition, a time-difference correction and window-stacking strategy is adopted to improve multi-channel arrival picking. Experimental validation on representative single-peak single-event and double-peak multi-waveform cases extracted from laboratory rock-failure tests demonstrates that the proposed method can effectively separate and identify AE waveforms under the tested conditions. Compared with conventional timing-parameter-based segmentation and correlation-dominated matching, the proposed workflow is more robust to waveform attenuation and distortion. The method provides a methodological basis for AE waveform identification and arrival-time extraction in rock-failure monitoring and has potential to support early warning after further validation.

## 1. Introduction

In recent years, with the continuous improvement of living standards, infrastructure construction has developed rapidly. However, various geotechnical engineering disasters accompany the construction process, such as slope instability, rock collapse, rockburst, and so on. These disasters cause significant casualties and economic losses and seriously threaten engineering safety. These disasters are typically macroscopic manifestations of internal fracture and instability within rock masses; therefore, understanding the evolutionary laws governing these processes is crucial for preventing and controlling such disasters [1,2,3]. Nevertheless, the mechanisms of rock fracture instability are complex. These mechanisms are closely related not only to the structural characteristics of rock, but also to environmental factors such as in situ stress and temperature, posing significant challenges to a comprehensive understanding of the mechanical properties of rock [4,5].

During rock failure, internal joint structures deform under stress, causing microcracks to propagate and gradually accumulate energy. When macrocracks form, the accumulated energy is rapidly released in the form of stress waves, resulting in dense acoustic emission (AE) signals [6,7,8]. These dense AE signals comprehensively reflect the dislocation and fracture of microscopic grains, the initiation and propagation of macroscopic cracks, and environmental factors associated with the rock in a specific state [9,10], thereby containing information regarding the evolution of the internal structure. Therefore, identifying dense AE signals during rock failure is conducive to a deeper understanding of the evolution mechanism of rock failure. This is essential for realizing dynamic monitoring, early damage warning, and failure prevention against rock destruction, and is of great significance for the prevention and control of geotechnical engineering disasters [11,12,13].

The identification of AE signals primarily involves two stages: waveform segmentation and waveform identification. Currently, waveform segmentation commonly relies on preset timing parameters defined by the Physical Acoustics Corporation (PAC) or Vallen Systeme to extract independent AE signals from data acquisition channels. These preset parameter-based waveform segmentation methods are straightforward and computationally efficient, and they perform well when AE events are sparse and waveform overlap is limited. However, under dense triggering conditions during rock failure, adjacent signals may arrive within the hit definition time (HDT), causing multiple events to be merged into one recorded waveform or truncated improperly. As a result, the segmentation performance of such methods may degrade significantly when signals are highly overlapping or strongly attenuated across channels. Regarding waveform identification, existing research can be primarily categorized into the source-scanning algorithm (SSA) and the matched filter method [14,15,16]. The former utilizes characteristics such as absolute amplitude, energy envelopes, or the short-term average/long-term average (STA/LTA) ratio to traverse potential source locations and origin times within the time series, thereby achieving waveform identification and localization [17,18,19,20]. Source-scanning-based methods determine event occurrence by scanning possible source locations and origin times in the monitored space and evaluating waveform coherence, energy stacking, or characteristic functions across channels. Their main advantage lies in the ability to detect and localize events in a multi-channel framework without requiring an exact waveform template for each event. However, such methods are often sensitive to waveform distortion, attenuation, and picking uncertainty, and their computational burden may become considerable when the search space is large or the event density is high. The latter, conversely, operates by calculating the cross-correlation between template waveforms recorded on each channel and the waveforms of potential AE events, subsequently using the superposition of correlated waveforms for identification [21,22]. Matched-filter-based methods are highly effective for detecting repeated or highly similar waveforms and are especially suitable for weak-event recovery in noisy data. Nevertheless, their performance strongly depends on the representativeness of the selected templates. When waveform deformation, arrival-time deviation, or missing-channel recording occurs due to attenuation or threshold-triggering effects, the matching reliability may decrease considerably [23,24]. In recent years, with the growth of dense/continuous monitoring data, machine-learning-based arrival picking, denoising, and event detection have also been increasingly introduced to improve robustness under low SNR and complex propagation conditions [25,26,27]. These methods show clear advantages in enhancing arrival-time consistency, reducing manual intervention, and improving weak-signal recognition. However, they usually require sufficiently labeled datasets for training, and their interpretability and transferability across different lithologies, loading paths, sensor layouts, and acquisition systems remain challenging [28,29,30].

However, AE signals generated by rock failure typically exhibit characteristics such as non-stationarity, large sample size, and high attenuation. During data acquisition, event triggering is extremely dense; if the interval between adjacent signals is less than the hit definition time (HDT), multiple events often overlap within the same time series, rendering methods based on preset timing parameters ineffective. Furthermore, once the signal exceeds the threshold and opens the channel, low-energy signals continue to enter, while some channels fail to record due to excessive signal attenuation. This results in inconsistent time series lengths across channels and significant deviations in the recorded arrival times of high-energy events. These phenomena cause misalignment, deformation, or loss of recorded waveforms, making it difficult for existing methods to effectively identify AE signals during the evolution of rock failure.

To address the aforementioned issues, this paper proposes a method for identifying AE signals. It should be noted that the novelty of the present study does not simply lie in combining multiple indicators, because multi-indicator analysis has already been widely used in AE research. Rather, the main contribution of this study is the development of an integrated identification workflow specifically for densely triggered and partially overlapping AE waveforms during rock failure. This workflow combines automatic waveform segmentation based on the waveform energy envelope, template-based sliding-window scanning across channels, joint evaluation of cross-correlation, ring count, rise time, and signal energy, and subsequent time-difference correction for improving arrival-picking consistency. Compared with existing multi-indicator studies that mainly use several parameters for event classification or qualitative discrimination, the proposed method is designed to address waveform overlap, attenuation-induced waveform deformation, inconsistent recorded lengths among channels, and arrival-time deviation within a unified and physically interpretable framework. First, the waveform energy envelope is employed to automatically segment individual waveforms within the time series. Subsequently, characteristic parameters such as waveform correlation, ring count, rise time, and signal energy are selected. A waveform template sliding window is then used to scan the time series across the acquisition channels, identify AE signals associated with rock failure, and apply time-difference correction to improve the accuracy of arrival picking for each event. This paper first introduces the evaluation indicators and analysis methods for AE signals, and then verifies the validity and localization accuracy of the proposed method by identifying and separating single-peak single-event and multi-peak waveform cases extracted from laboratory rock-failure tests.

## 2. Waveform Segmentation Method Based on the Waveform Energy Envelope

AE signals are triggered densely; consequently, each acquisition channel may simultaneously contain multiple AE signals with extremely short intervals between preceding and succeeding waveforms. Methods based on PAC and Vallen preset timing parameters might fail to effectively segment these individual waveforms. Furthermore, due to the rapid attenuation of AE waveforms, amplitudes in certain channels may fall below the threshold and remain unrecorded, leading to inconsistent time-series lengths and significant deviations in observed arrival times. Therefore, to achieve effective separation of dense waveforms in AE, this study adopts a method that uses the waveform energy envelope to automatically segment waveforms within the time series.

The morphology of AE waveforms may vary with phase. Therefore, it is initially necessary to calculate the envelope of the time series within the channels to eliminate phase effects, using it to approximate energy variations. The Hilbert transform is employed to calculate the envelope, considering this integral as the Cauchy principal value, which avoids the singularity at τ=t:

(1) $$X\left(t\right)=x\left(t\right)*h\left(t\right)=\int_{0}^{M}x\left(\tau \right)h\left(t-\tau \right)d\tau =\frac{1}{\pi }\int_{0}^{M}\frac{x\left(\tau \right)}{t-\tau }d\tau$$

where $X\left(t\right)$ is the envelope waveform, $x\left(t\right)$ is the waveform, and $h\left(t\right)=\frac{1}{\pi t}$.

**Figure 1:**
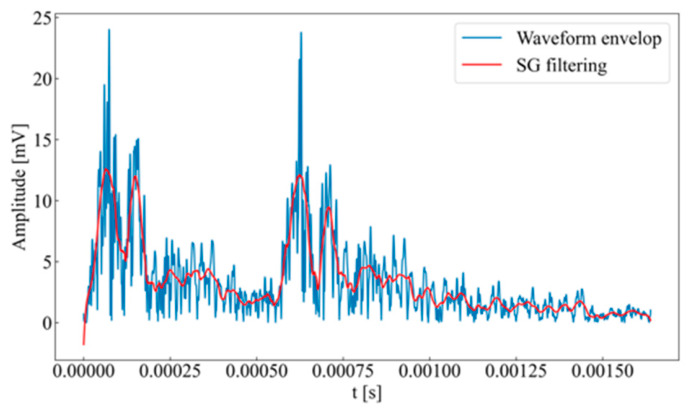
Waveform envelope and its SG filtering.

The signal derived from the envelope calculation exhibits instability, which is unfavorable for waveform segmentation; therefore, additional smoothing is required. A Savitzky–Golay (SG) filter [31], based on local polynomial fitting of curves, is employed. This filtering method uses least squares to determine the weighting coefficients for a moving-window weighted average. It ensures that the reconstructed data effectively preserve the local characteristics of rock acoustic emission events and better reflect the variation in signal amplitude. The results of the calculation are presented in Figure 1.

**Figure 2:**
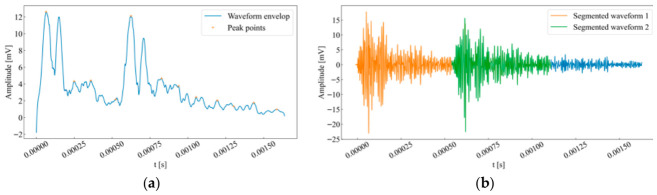
(**a**) Waveform envelope and its distribution of peak points. (**b**) Demonstration of the waveform segmentation method.

The energy of the main body of an AE signal is significantly higher than that of precursor waves and background noise. Therefore, by searching for and screening the peak points within the filtered time series (as shown in Figure 2a), it is possible to preliminarily determine the number of potential rock AE events.

In this study, the waveform envelope was smoothed using a Savitzky–Golay filter with a window length of 901 samples and a polynomial order of 5. Peaks were screened from the filtered envelope with a minimum spacing of 1000 samples. Only peaks above 50 dB and within 5 dB of the maximum peak were retained. For the single-peak case, the onset was defined from the last pre-peak threshold-crossing point with a backward extension of 500 samples (0.05 μs), and the termination point was taken as the first post-peak sample where the envelope decayed to 20% of the maximum amplitude. For the multi-peak case, the separation point was defined as the minimum envelope value between adjacent retained peaks, and the termination point of the subsequent waveform was taken as the first post-peak sample where the envelope decayed to 10% of the maximum amplitude. The resulting waveform segmentation is illustrated in Figure 2b.

## 3. Characteristic Parameters for AE Signal Identification

AE signals predominantly exhibit nonlinear and non-stationary characteristics. Generated by distinct micro-fractures during rock failure, AE signals exhibit substantial differences due to variations in loading type (tension, shear, and torsion) and magnitude. These distinctions are specifically manifested in characteristic differences regarding cross-correlation coefficient, frequency, energy, and strength. Conversely, although signals originating from the same micro-fracture source may vary in propagation paths, they retain a high degree of similarity in the aforementioned characteristic parameters. Leveraging these signal discrepancies allows for the effective identification and classification of AE signals associated with different micro-fracture sources, loading types, and magnitudes.

The four characteristic parameters adopted in this study were selected based on their physical meanings and complementary discrimination capabilities. Specifically, the cross-correlation coefficient is used to quantify waveform-shape similarity, ring count reflects oscillatory and threshold-crossing behavior, rise time characterizes onset development and propagation-related broadening, and signal energy reflects the overall intensity of the waveform body. These parameters describe different but complementary aspects of AE waveforms. Therefore, their combined use can improve the robustness of AE event identification under conditions of dense triggering, waveform overlap, attenuation, and partial waveform distortion.

### 3.1. Cross-Correlation Coefficient

The cross-correlation coefficient typically indicates the degree of correlation between the variations of two waveforms. In this paper, the cross-correlation coefficient was used to quantify the similarity between the scanned waveform within a sliding time window and a template waveform. Assuming two waveforms denoted as x(t) and y(t), the cross-correlation coefficient between them is expressed as:

(2) $$CC\left(i\right)=\left|\frac{\sum_{t=1}^{M}x\left(t\right)y\left(t\right)}{\sqrt{\sum_{t=1}^{N}x^{2}\left(t\right)\sum_{t=1}^{N}y^{2}\left(t\right)}}\right|,i\in 1,2,\ldots ,M-N+1$$

where $CC\left(i\right)$ represents the cross-correlation coefficient of the sampling points corresponding to the sliding time window, N is the total number of sampling points within the sliding time window, i is the starting point of the sliding time window, and M is the total number of sampling points in the time series recorded within the channel.

Let the waveform within the time window to be identified be denoted as $x\left(t\right)$, which is then subjected to cross-correlation calculation with the template waveform $y\left(t\right)$. The value range of the cross-correlation coefficient is [0, 1]. The magnitude of this coefficient reflects the correlation of the amplitude variation trends between the scanned waveform and the template waveform: the higher the similarity, the closer the value approaches 1; conversely, the higher the proportion of signals from different micro-fracture sources or random noise contained within the window, the closer the value approaches 0.

The cross-correlation coefficient method can effectively address the difficulty of identifying successive waveforms with excessively short intervals within the same time series. When the cross-correlation coefficient for the sliding time window exceeds a preset threshold, it can be inferred that the waveform recorded within the window and the template waveform belong to an acoustic emission event triggered by the same micro-fracture source.

However, the cross-correlation coefficient primarily reflects the consistency of waveform variation trends and is sensitive to waveform distortion and energy attenuation. Therefore, it is typically necessary to integrate other identification indicators to achieve the effective identification of AE signals.

### 3.2. Ring Count

Ring count is the number of times a signal oscillates across a preset threshold, reflecting the frequency characteristics of the scanned waveform within a sliding time window. Due to its simplicity in processing, this metric is widely used to evaluate the activity of AE signals. As an external acoustic response to changes in the internal joint structure of rock, ring count embodies the intensity of AE activity and the evolutionary process of internal rock damage; their values are influenced by factors such as elastic modulus, initial defects, and rock structure [4,32]. Since AE signals originating from the same micro-fracture source exhibit a certain degree of similarity in ring count, this metric can serve as one of the criteria for identifying AE signals. In this paper, ring count was adopted to measure the average frequency of the waveform within the sliding time window, with the calculation formula as follows:

(3) $$C\left(i\right)=\frac{n}{N},i\in 1,2,\ldots ,M-N+1$$

where $C\left(i\right)$ represents the ring counts of the sampling points corresponding to the sliding time window, n denotes the number of times the signal crosses the threshold within the sliding time window, N is the total number of sampling points within the sliding time window, and M is the total number of sampling points in the time series recorded within the channel.

### 3.3. Rise Time

Rise time is defined as the time interval from the moment an AE signal first crosses the threshold to the point where it reaches its maximum amplitude. It is widely used in research on fracture mode identification and noise signal discrimination. Rise time reflects the influence of rock properties on wave propagation. During propagation, influenced by dispersion and attenuation, signals near the source exhibit shorter rise times. Conversely, for signals distant from the source, the rise time is relatively longer due to the rapid attenuation of high-frequency components and peak amplitudes.

### 3.4. Signal Energy

Signal energy is a parameter closely associated with signal amplitude and its distribution, typically defined as the area under the AE signal envelope. Although this parameter primarily has mathematical significance and does not correspond to the true physical energy of the AE signal, it effectively reflects the relative energy intensity of the event and is important for assessing the degree of rock rupture and damage. Waveforms originating from different micro-fracture sources exhibit significant differences in signal energy. Furthermore, the signal energy of the waveform’s main body is distinct from that of background noise, thereby facilitating signal identification and separation. The calculation formula is as follows:

(4) $$I\left(i\right)=\sum_{t=1}^{N}X\left(t\right),i\in 1,2,\ldots ,M-N+1$$

where $I\left(i\right)$ represents the signal energy of the sampling points corresponding to the sliding time window, and $X\left(t\right)$ denotes the energy envelope of the scanned waveform $x\left(t\right)$ within the sliding window. Signal energy is unaffected by the threshold or phase, making it applicable for the identification of AE signals.

In this study, these indicators are not used independently, but are integrated within a waveform-template sliding-window framework. Their joint use is intended not only to improve waveform discrimination, but also to support dense time-series scanning and subsequent arrival correction. Therefore, the proposed method differs from conventional multi-indicator discrimination approaches that mainly focus on post-event classification or qualitative comparison, because here the selected indicators are embedded directly into the AE waveform identification process itself.

## 4. The Multi-Indicator Fusion-Based Technique for Identification and Correction of AE Signals

### 4.1. Template Channel Selection

Rock micro-fractures are influenced by variations in loading type and magnitude, resulting in significant diversity in the induced AE event signals. Reliance solely on individual typical waveforms as templates for scanning is highly prone to causing the misidentification and omission of events. To address this issue, this paper used actual waveforms recorded in the acquisition channels as templates and employed a sliding-scan method for processing.

The length of the unit time series for each channel was set to 5000 μs. To avoid ambiguity when SNR and signal energy are inconsistent, the template channel was selected using a combined score based on normalized SNR and normalized signal energy:

(5) $$Score_{j}={S\hat{N}R}_{j}\times {\hat{E}}_{j}$$

where ${S\hat{N}R}_{j}$ and ${\hat{E}}_{j}$ are the normalized SNR and normalized signal energy of channel j, respectively. The channel with the maximum combined score was selected as the template channel. When competing channels had very similar scores, priority was given to the one with the higher SNR to reduce noise interference and ensure waveform stability. Waveforms extracted from the selected template channel were then used for sliding-window scanning in the remaining channels.

This strategy avoids matching errors caused by preset fixed templates and improves adaptability to temporal variations in source location, waveform attenuation, and channel quality.

### 4.2. Sliding Window Scanning

The identification of AE signals is a dynamic process; therefore, the analysis of unit time series must be conducted within specific time windows. The selection of the time window length is critical. If the window is too long, it not only increases computational time and reduces identification efficiency but may also result in the inclusion of multiple overlapping event signals within a single window. Conversely, if the window is too short, it may fail to fully cover the waveform, potentially causing a single event to be misclassified as two adjacent events, thereby diminishing identification accuracy. Furthermore, because micro-fracture scales vary, the duration of each AE signal also varies; consequently, employing a fixed-length time window is detrimental to efficient event identification.

To address this, this paper proposes an adaptive adjustment strategy. First, the approximate region where an AE signal may exist is extracted based on the waveform energy envelope method. Subsequently, using this region as a baseline, the window is extended forward and backward by one window length each. The total length of these three continuous windows is established as the sliding scan interval for the waveform. This method allows for timely adjustment of the window length during the automatic identification process to match the actual micro-fracture scale, thereby accommodating waveforms of varying duration.

**Figure 3:**
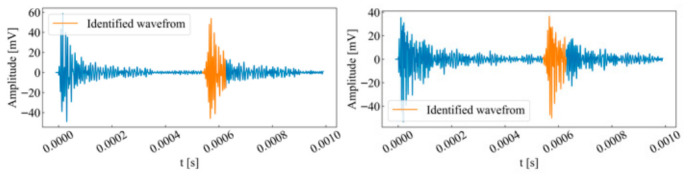
Demonstration of window scanning based on the template channel.

Once the sliding scan interval is determined, it is necessary to calculate and compare identification indicators between the template waveform and the waveform within the scanning window. Whether to introduce the SNR as an indicator depends on the intrinsic SNR level of the unit time series: if the series possesses a high SNR, this indicator need not be considered; otherwise, it must be incorporated into the evaluation system. Finally, the similarity coefficient for each window corresponding to a sampling point within the sliding scan interval is calculated, and the window with the maximum value is selected as the best match. The calculation formula for the similarity coefficient $SC\left(i\right)$ is as follows:

(6) $$SC\left(i\right)=CC\left(i\right)*\frac{\left|C\left(i\right)-C^{\prime }\right|}{C^{\prime }}*\frac{\left|RA\left(i\right)-RA^{\prime }\right|}{RA^{\prime }}*\frac{\left|I\left(i\right)-I^{\prime }\right|}{I^{\prime }},i\in 1,2,\ldots ,M^{\prime }-N+1$$

where $C^{\prime }$, $RA^{\prime }$, and $I^{\prime }$ represent the ring counts, rise time, and signal energy of the template waveform, respectively, and $M^{\prime }$ denotes the number of sampling points within the sliding scan region. The schematic diagram of the template scanning process is shown in Figure 3. Compared with the cross-correlation coefficient, the similarity coefficient avoids misidentification caused by similarities in fluctuations between noise and the template waveform within shorter time windows. Furthermore, integrating multiple identification indicators is more effective for identifying waveforms that have undergone deformation, attenuation, or dislocation during propagation.

### 4.3. Time-Difference Correction

The time-difference correction flattens the arrivals of potential micro-fracture signals within the records, thereby enabling them to exhibit high consistency across each unit time series. If the series contains only random noise, the channel records will remain as noise following time-difference correction. Upon obtaining the sampling points corresponding to the maximum similarity coefficients, they are designated as $t_{i}$, and the relative time difference for each channel’s best-matching window is calculated. However, due to the varying propagation paths of rock acoustic emission events to different sensors, as well as the influence of factors such as noise and attenuation, waveforms of the same acoustic emission event recorded across various acquisition channels can differ. Therefore, it is necessary to construct the following linear equation:

(7) $$\left[\begin{array}{cccc} \begin{array}{c} 1\\ 1 \end{array} & \begin{array}{c} -1\\ 0 \end{array} & \begin{array}{c} 0\\ -1 \end{array} & \begin{array}{c} 0\\ 0 \end{array}\\ \begin{array}{c} 1\\ 0 \end{array} & \begin{array}{c} 0\\ 1 \end{array} & \begin{array}{c} 0\\ -1 \end{array} & \begin{array}{c} -1\\ 0 \end{array}\\ \begin{array}{c} 0\\ 0 \end{array} & \begin{array}{c} 1\\ 0 \end{array} & \begin{array}{c} 0\\ 1 \end{array} & \begin{array}{c} -1\\ -1 \end{array}\\ 1 & 1 & 1 & 1 \end{array}\right]\left[\begin{array}{c} t_{1}\\ t_{2}\\ t_{3}\\ t_{4} \end{array}\right]=\left[\begin{array}{c} \begin{array}{c} \Delta t_{12}\\ \Delta t_{13} \end{array}\\ \begin{array}{c} \Delta t_{14}\\ \Delta t_{23} \end{array}\\ \begin{array}{c} \Delta t_{24}\\ \Delta t_{34} \end{array}\\ 0 \end{array}\right]$$

It can be abbreviated as the following form:

(8) At=Δt

where A is the sparse coefficient matrix, Δt is the relative time-difference vector, and t is the unknown time-difference correction parameter vector. Using the least squares solution, we obtain:

(9) $$t={\left(A^{T}A\right)}^{-1}A^{T}\Delta t$$

Once t is solved using the above equation, time-difference correction can be performed on the records of each channel based on the sampling points $t_{i}$ corresponding to the maximum values of the similarity coefficients. Subsequently, the windows are stacked. Since the waveforms recorded in the respective windows exhibit high consistency while the random noise is mutually uncorrelated, the SNR of the resulting stacked channel is higher than that of the individual channel records prior to stacking.

### 4.4. Arrival Picking

This paper employed the Akaike information criterion (AIC) [33] for arrival picking. The AIC picker assumes that the time windows before and after the arrival correspond to two different stationary time series. For an AE signal $x\left(t\right),t=1,2,\ldots N$, the AIC value is given as a function of the merging point k:

(10) $$\mathrm{AIC}\left(k\right)=\left(k-M\right)*\log\left(\sigma_{1,\max}^{2}\right)+\left(N-M-k\right)\log\left(\sigma_{2,\max}^{2}\right)+C_{2}$$

where M is the order of the autoregression for the fitted data; $\sigma_{1,\max}^{2}$ and $\sigma_{2,\max}^{2}$ represent the variances of the prediction error for the time series within the intervals $\left[M+1,k\right]$ and $\left[k+1,N-M\right]$, respectively; and $C_{2}$ is a constant. To obtain the AIC value, the autoregressive coefficients and the order M must be calculated, both of which involve high computational complexity. In contrast to the aforementioned AIC picker, the AIC function can be calculated directly from the seismogram without using autoregressive coefficients. This AIC function is defined as:

(11) $${\mathrm{AIC}}_{n}=n\mathrm{lg}\left(Var\left[U_{n-}\right]\right)+\left(N-n-1\right)\mathrm{lg}\left(Var\left[U_{n+}\right]\right)$$

where $U_{n-}$ and $U_{n+}$ represent the signals preceding and succeeding point n, respectively. While the AIC method can be highly accurate for seismic signals with distinct P-phases, the AIC picker may exhibit significant errors when applied to seismic signals with relatively low signal-to-noise ratios. Therefore, it is essential to incorporate the AIC method into multi-scale analysis to mitigate noise.

## 5. Experiments

The AE waveforms analyzed in this study were obtained from laboratory loading tests on granite specimens. The tests were carried out using a TRW-3000 true triaxial electrohydraulic servo system. AE monitoring was performed using an AMSY-6 multi-channel AE system with a sampling rate of 10 MHz. VS45-H AE sensors (Vallen Systeme GmbH, Wolfratshausen, Germany), with a frequency response range of 20–450 kHz, were mounted on the specimen surfaces using a coupling agent and fixtures to ensure stable signal transmission. The acquired AE waveform data were used for waveform segmentation, signal identification, and arrival-time correction in this study.

### 5.1. Segmentation and Identification of Single-Peak Single-Waveforms

**Figure 4:**
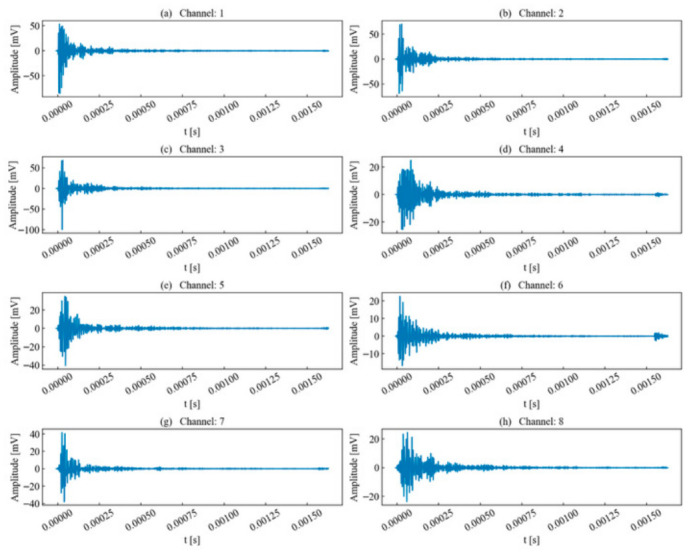
Channels to be identified for single-peak single-waveforms.

This paper first selected the typical time series of single-peak single-events extracted from continuous AE records acquired during laboratory rock-failure tests for analysis. Such waveforms are generally generated by a single micro-fracture source, where the excitation time interval between adjacent micro-fracture sources exceeds the duration of a typical signal. The unit time series to be analyzed is illustrated in Figure 4. During propagation, the AE waveforms exhibit a certain degree of attenuation, deformation, and elongation.

(1) Selection of template channel

Prior to waveform segmentation and identification, the template channel must be selected based on the SNR and signal energy of each unit time series. To determine the noise signal power, the first 500 sampling points of each unit time series were used as background noise, and their power is computed. Subsequently, the signal power for each acquisition channel is calculated, and the logarithm of the ratio between the signal power and the noise power is taken to obtain the SNR for each channel.

The following table lists the SNR and signal energy for each acquisition channel. As indicated in the table, the overall SNR of the unit time series is favorable, with a minimum value greater than 9. Notably, Acquisition Channel 2 exhibited the highest SNR, approximately SNR=20.82.

In addition to SNR, signal energy serves as a crucial criterion for selecting the template channel. Since the signal energy of AE signals is significantly higher than that of noise signals, and waveforms with less attenuation typically exhibit greater signal energy, it is rational to select the sequence with the maximum signal energy as the template channel. As shown in Table 1, the signal energy of Acquisition Channel 2 was approximately twice that of the weaker channels.

Although a comparison revealed a consistency in the ranking of SNR and signal energy across channels, relying solely on a single indicator may lead to the erroneous selection of a sequence with, for instance, low SNR but high intensity (or vice versa), thereby compromising the efficacy of subsequent waveform identification. Therefore, a comprehensive consideration of both indicators is imperative. In summary, for this unit’s time series, Acquisition Channel 2 was selected as the template channel for waveform identification in other acquisition channels.

(2) Waveform segmentation

**Figure 5:**
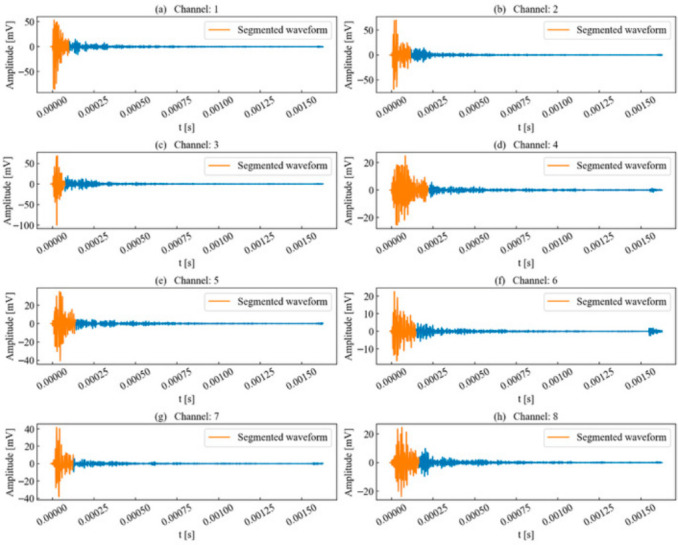
Waveform segmentation of single-peak single-waveforms.

The waveform energy envelope method was applied to segment the waveforms for each unit time series, with the results shown in Figure 5. Due to attenuation, the waveforms segmented in certain channels were significantly elongated compared to the template channel. This elongation occurs because the decrease in envelope energy causes the termination point, which serves as the segmentation benchmark, to shift backward.

Nevertheless, compared to the preset timing parameter method based on amplitude, the waveform energy envelope method is less susceptible to the influence of noise signals. As shown in Figure 5, channels with significant signal attenuation still exhibit amplitude fluctuations after the termination point. If an amplitude threshold is used as the benchmark, these fluctuations might be erroneously classified as part of the waveform. However, from an energy perspective, the waveform envelope energy at this stage has stably decayed below the threshold, meeting the segmentation requirements and thereby effectively avoiding interference from subsequent noise signals on the waveform.

(3) Waveform Identification

Using the template channel, a sliding-window scan was performed over the scanning intervals of each channel to calculate the corresponding rise time ratio, ring count ratio, signal energy ratio, and similarity coefficient. From these, the best matching window was selected.

**Figure 6:**
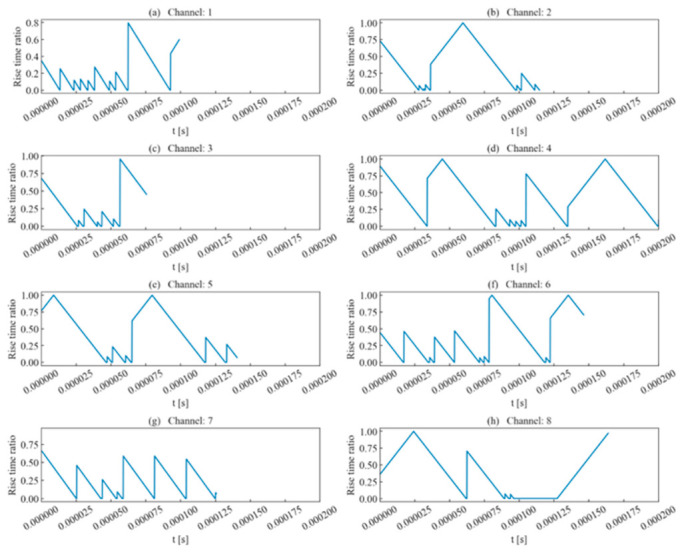
Rise time ratio of single-peak single-waveforms.

As shown in Figure 6, using rise time as a judgment criterion showed some effectiveness, with results that remained consistent with the waveform’s approximate region. For certain sliding windows, if the ratio was negative (indicating an excessive deviation from the template waveform’s rise time), this paper set the value to 0 to prevent interference with subsequent analysis.

**Figure 7:**
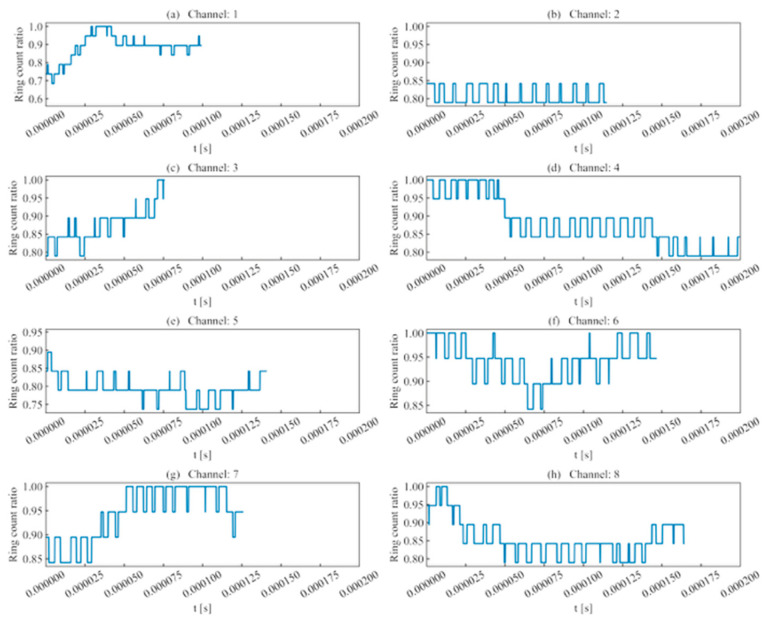
Ring count ratio of single-peak single-waveforms.

Regarding ring counts, as shown in Figure 7, the difference in ring count ratios across the analyzed unit time series was no greater than 0.2. This indicates that for AE signals, although high-frequency information is attenuated, the magnitude loss is minimal, and waveform deformation primarily stems from amplitude attenuation.

**Figure 8:**
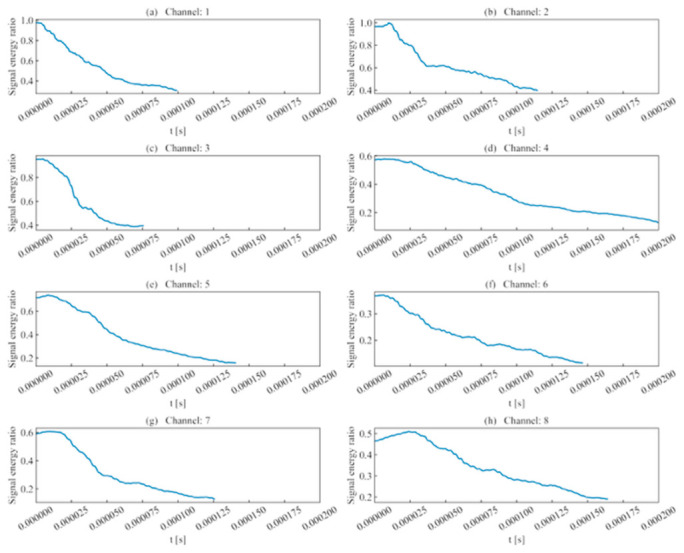
Signal energy ratio of single-peak single-waveforms.

In terms of signal energy, as shown in Figure 8, since the template channel was selected as the signal with the maximum intensity among all channels, the signal energy ratios obtained from scanning other channels mostly fell within [0, 1]. Observation of the channels reveals that when scanning regions of high signal energy, the signal energy ratio for a complete waveform is relatively high; however, as the window passes the peak sampling point and includes low-intensity noise, this ratio gradually decreases.

**Figure 9:**
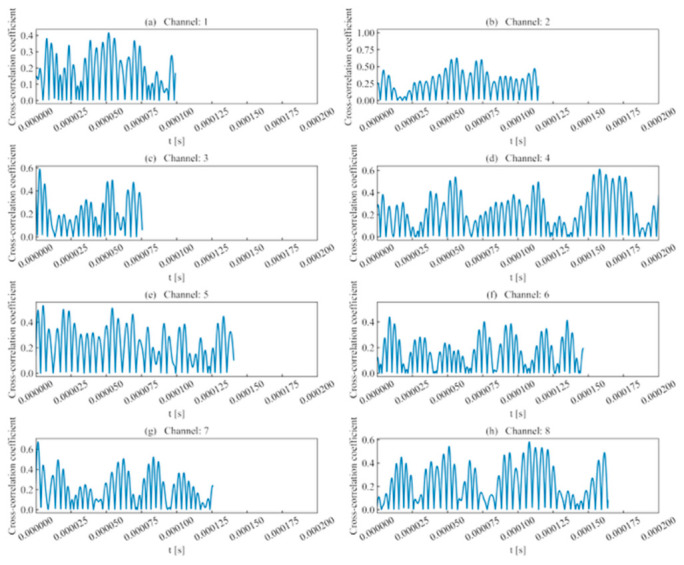
Cross-correlation coefficient of single-peak single-waveforms.

In channels with low signal intensity, the overall signal energy is low due to waveform energy attenuation during propagation. Although the sliding scan still exhibited a characteristic higher signal energy before the peak and a lower ratio thereafter, the fluctuation was relatively gentle and susceptible to noise interference. As shown in Figure 9, the cross-correlation coefficient fluctuated significantly as the sliding window moved. This is because the cross-correlation coefficient primarily reflects the similarity in amplitude fluctuations; however, the severe attenuation of AE signals leads to significant waveform distortion, making it difficult to make accurate judgments based solely on the cross-correlation coefficient.

**Figure 10:**
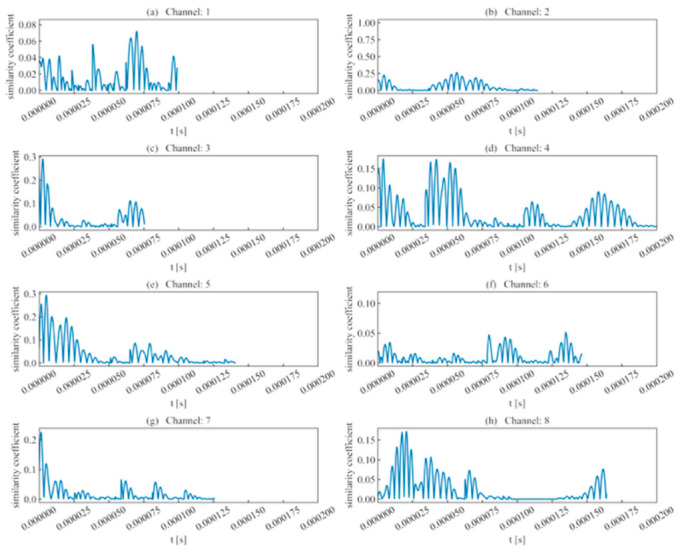
Similarity coefficient of single-peak single-waveforms.

Recognizing that individual characteristic parameters capture different aspects of the signal, this study defines the similarity coefficient as a waveform-identification metric, computed as the product of these parameters. As illustrated in Figure 10, for acquisition channels with high SNR and high signal intensity, the maximum similarity coefficient is accurately near the waveform’s actual region, whereas for noise signals, this coefficient approaches zero.

**Figure 11:**
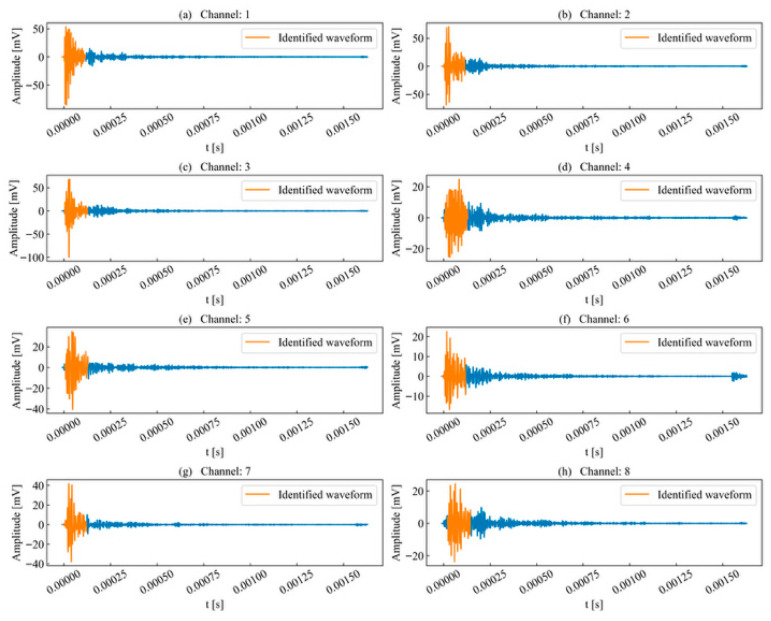
Identification results of single-peak single-waveforms.

Based on the similarity coefficient, the most similar waveforms for each channel were extracted (as shown in Figure 11). The results indicate that under this index, the sliding window accurately matches the waveform’s main body in each channel, well covering the onset, peak, and termination points. Although the waveforms in some channels exhibited a certain degree of distortion and energy attenuation relative to the template waveform, the method proposed in this paper effectively mitigates the effects of propagation attenuation, enabling accurate waveform identification within each channel.

(4) Arrival picking and correction

To perform time-difference correction, the arrival within the most similar window was selected as the reference point. The least-squares method was used to calculate the time-difference correction values for each window. Translation was achieved by differencing the time series axes, and the stacking range was determined by the extreme values of the left and right boundaries of each window. The figure below displays the stacking windows for each unit event sequence; the length of the window after time-difference correction was greater than that of the original most similar window.

Stacking processing was performed on each corrected window. Since noise signals are random and uncorrelated, while AE waveforms originating from the same micro-fracture source possess similarities in characteristic parameters and waveform fluctuations, the in-phase axes of the waveforms are flattened after time-difference correction. Consequently, within the stacking window, noise signals are attenuated by phase differences, while the effective AE signals are enhanced, thereby significantly improving the SNR.

**Figure 12:**
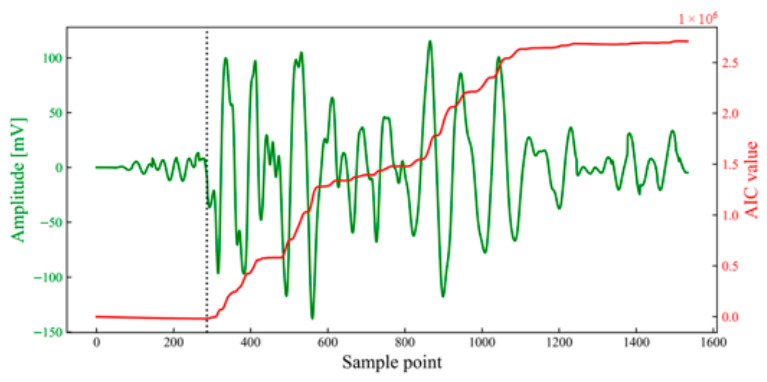
Demonstration of arrival picking for single-peak single-waveforms. The dotted line indicates the sample point corresponding to the arrival determined based on the AIC value.

**Figure 13:**
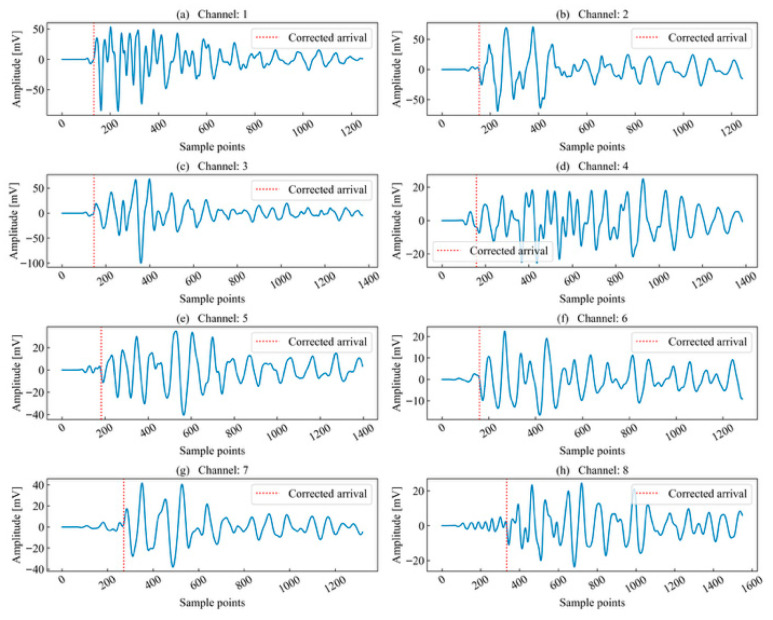
Arrival correction of single-peak single-waveforms.

Subsequently, arrival picking was performed on the stacked window to obtain the arrival under high SNR conditions (as shown in Figure 12). By back-calculating using the time-difference correction values, the original arrival times in each unit time series can be obtained (as shown in Figure 13 and Table 2). The results demonstrate that the determined arrival times for each channel are located substantially near the onset point. This extraction and back-calculation method based on stacking windows is not only capable of accurately picking arrival times for high-SNR and high-energy waveforms, but also demonstrates good applicability for waveforms with significant attenuation and distortion.

To quantitatively evaluate the improvement in arrival-time extraction, the absolute value of the sample correction introduced by time-difference correction was normalized by the total number of samples in a single-channel record and used as an indicator of the improvement after correction, termed the correction ratio CV. The average CV of the results was 5.3125%.

### 5.2. Segmentation and Identification of Double-Peak Multi-Waveforms

**Figure 14:**
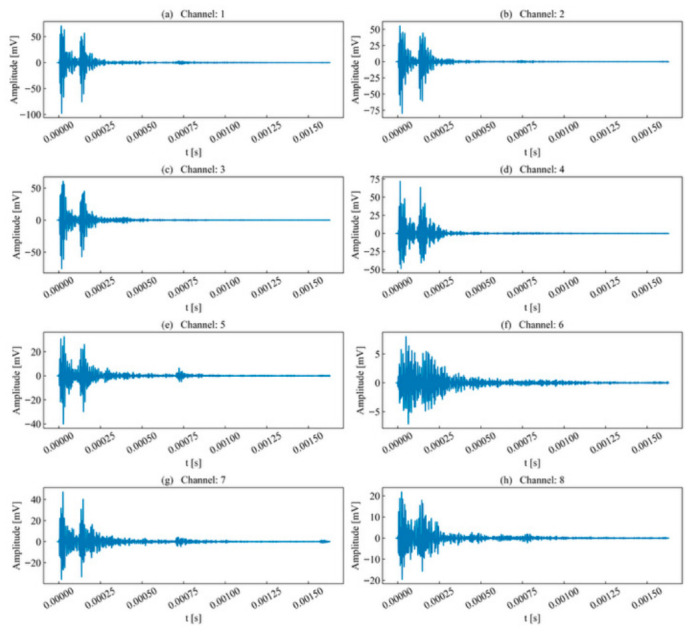
Channels to be identified for double-peak multi-waveforms.

In this section, the time series exhibiting typical multi-peak characteristics extracted from continuous AE records acquired during laboratory rock-failure tests was selected for analysis, as illustrated in Figure 14. Within this sequence, two AE signals possessed similar signal intensities and were separated by a short temporal interval. Typically, the duration of a single signal is approximately 1000 μs. However, because the subsequent event enters the channel before the preceding event has fully concluded, the coda wave signal of the first signal fails to decay sufficiently to meet the segmentation criteria defined by preset timing parameters. Consequently, the two waveforms overlap in the same channel. This sequence was selected to validate the effectiveness of the proposed method in segmenting and identifying multi-peak, multi-waveform signals. Here, the multi-peak waveform case was treated as a representative short-interval overlapping waveform case for the methodological validation of waveform segmentation and identification.

(1) Selection of template channel

First, the template channel is selected by calculating the SNR and energy for each channel; the results are presented in Table 3. Overall, the SNR of each channel is favorable, with a minimum value exceeding 16. The first channel exhibited the highest SNR, approximately SNR=34.0. Regarding signal energy, the first channel was roughly four times stronger than the weaker channels. Thus, the first channel was selected as the template channel for identifying waveforms in other acquisition channels.

(2) Waveform segmentation

**Figure 15:**
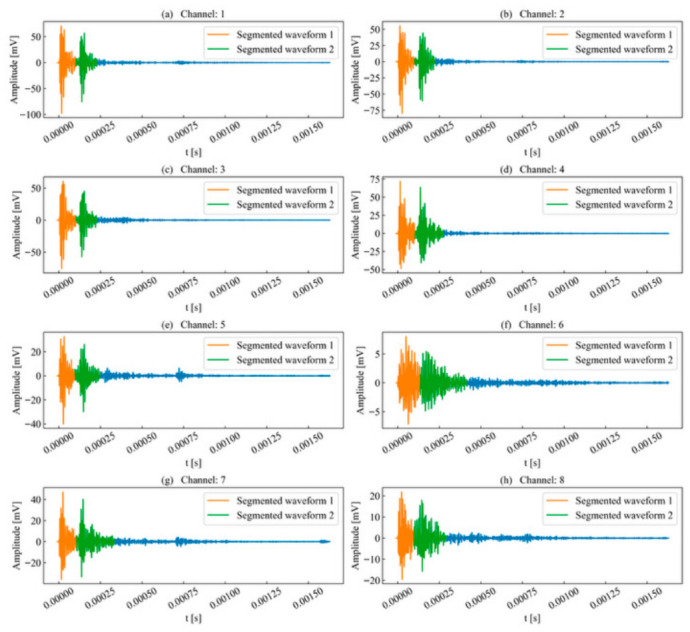
Waveform segmentation of double-peak multi-waveforms.

Subsequently, the waveform energy envelope method was applied to each acquisition channel, and the results are shown in Figure 15. The results indicate that this method effectively segments unit time series that cannot be processed by PAC and Vallen preset parameters. The distinction between preceding and succeeding signals in each sequence is clear; despite a certain degree of overlap, the termination point of the preceding signal and the onset point of the succeeding signal can still be reasonably determined through fluctuations in the waveform energy envelope.

In high-SNR channels, waveform energy fluctuations are significant; as energy decays, the overall amplitude decreases. In low-SNR channels, the changes in energy fluctuation are more gradual; for instance, although the boundary between two events in Channel 6 was indistinct, the method still achieved effective segmentation. The termination point of a waveform is determined based on the stable decay of the peak amplitude energy to a specific percentage threshold. For high signal energy channels, the termination point of the succeeding event falls before 250 μs; whereas for low signal energy channels, the distance between the peak and the termination point of the succeeding event is elongated due to the decrease in peak amplitude. Although the lengths of the segmented waveforms vary across channels, the template channel was selected based on the principle of maximum SNR and signal energy, yielding the shortest event length among the corresponding events. This study used the length of the template channel as the sliding time window length and the length of the corresponding event in other sequences as the scanning interval. Consequently, the elongated distance in low signal energy channels does not affect the search for the most similar event.

(3) Waveform identification

Using the template channel, a sliding-window scan was performed on each unit event sequence to compute the rise time ratio, ring count ratio, signal energy, cross-correlation coefficient, and similarity coefficient within the scanning interval. The results are illustrated in the figures below.

**Figure 16:**
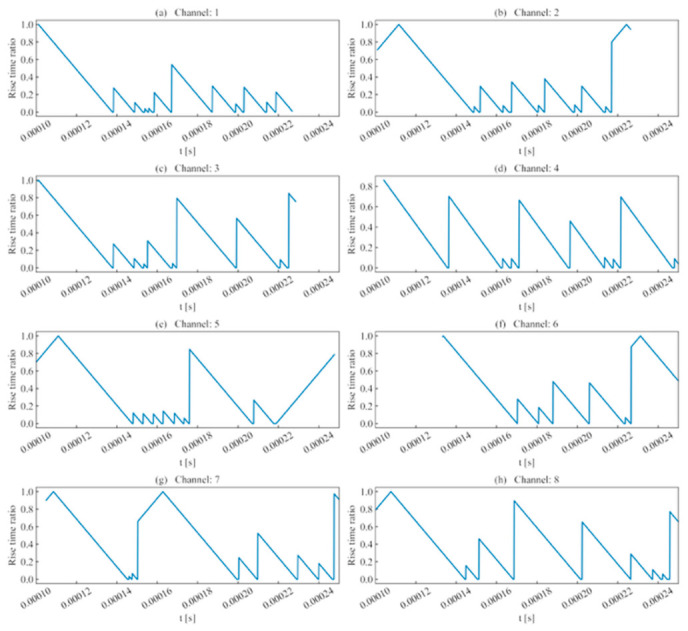
Rise time ratio of double-peak multi-waveforms.

**Figure 17:**
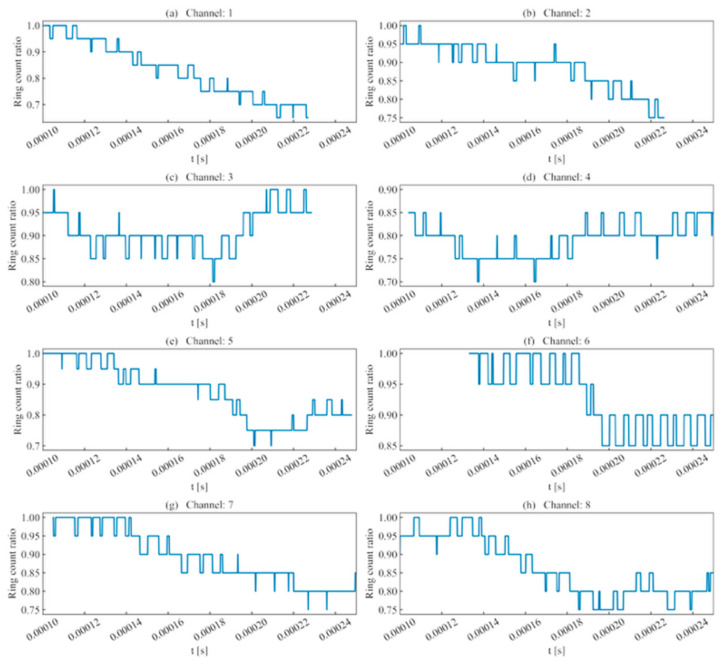
Ring count ratio of double-peak multi-waveforms.

**Figure 18:**
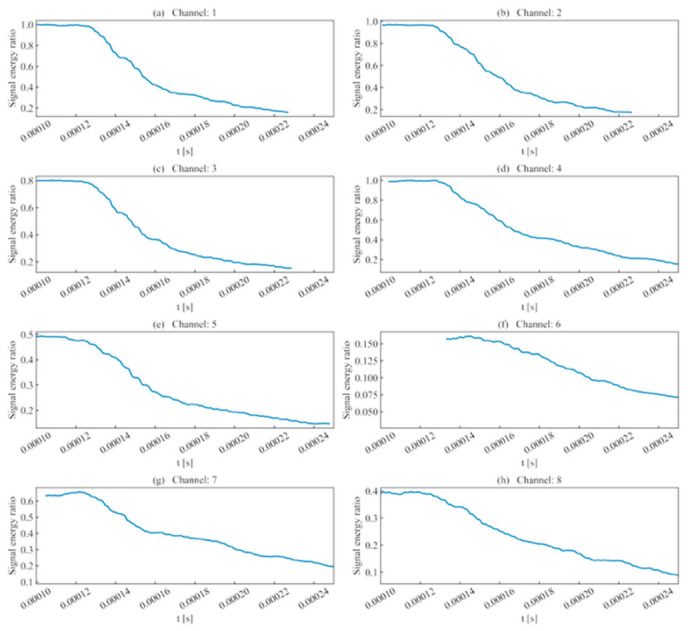
Signal energy ratio of double-peak multi-waveforms.

**Figure 19:**
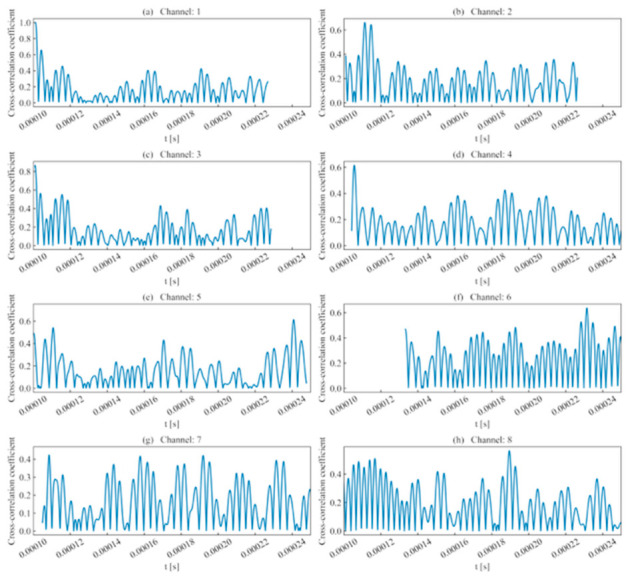
Cross-correlation coefficient of double-peak multi-waveforms.

(1) Rise time ratio (Figure 16): High rise time ratios in each channel are primarily distributed near 10 μs, showing consistency with their actual distribution regions.
(2) Ring count ratio (Figure 17): The difference in ring count ratios across unit event sequences is no greater than 0.2.
(3) Signal energy ratio (Figure 18): Within the same channel, the signal energy ratio gradually decreases as the window slides past the peak sampling point and incorporates weaker noise signals. Across different channels, the maximum values of the ratios vary due to signal attenuation during propagation.
(4) Cross-correlation coefficient (Figure 19): A comparison with signal energy reveals that channels with high signal energy ratios exhibit a gentler trend in cross-correlation coefficient changes, while those with low signal energy ratios show a sharper trend. This may be attributed to the increased frequency of zero-crossings and accelerated waveform fluctuations caused by the decrease in waveform amplitude. This indicates that for waveforms with high SNR and signal energy (e.g., Channels 1, 3, and 5), the cross-correlation coefficient meets the identification requirements. However, for low-SNR, low-intensity waveforms, the cross-correlation coefficient exhibits numerous local maxima, making it difficult to use as the sole basis for identification.

**Figure 20:**
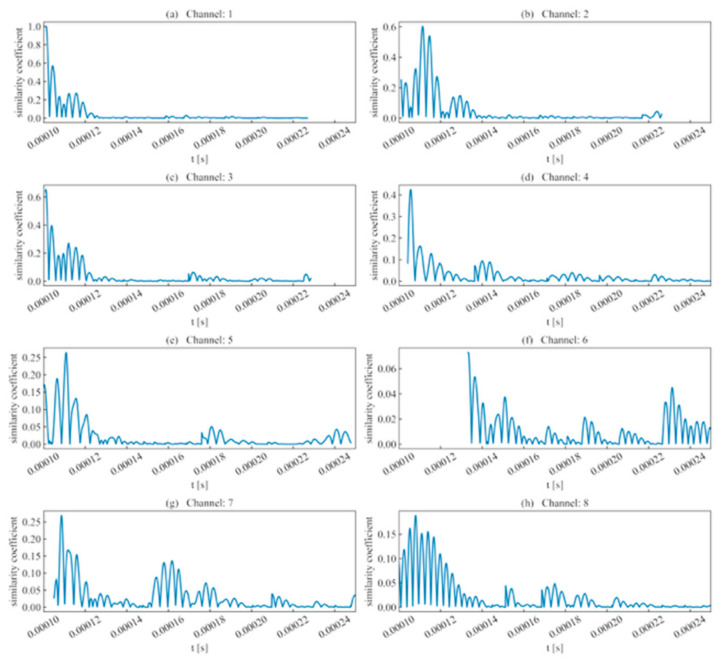
Similarity coefficient of double-peak multi-waveforms.

In light of these circumstances, this study adopted the similarity coefficient for waveform identification, as shown in Figure 20. The results demonstrate that after correction using waveform characteristic parameters such as rise time and ring count, the local maxima points in the similarity coefficient are significantly reduced, which is of great significance for selecting the most similar waveform.

**Figure 21:**
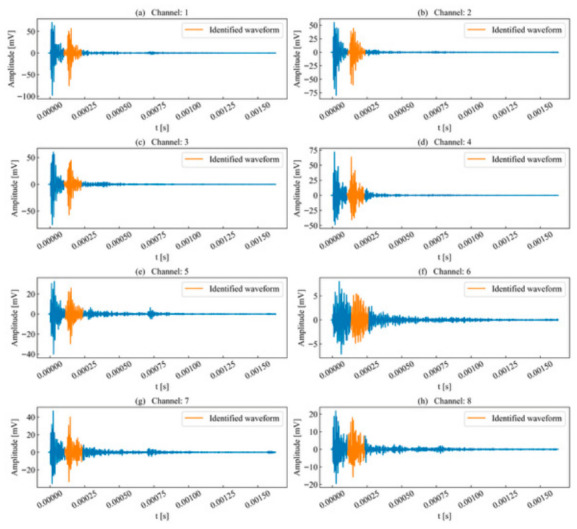
Identification results of double-peak multi-waveforms.

Based on the calculated similarity coefficients, the most similar windows for each acquisition channel were determined, as shown in Figure 21. The results indicate that the template waveform can accurately match corresponding waveforms in most unit time series and effectively covers the onset, peak, and termination points of each waveform. Due to variations in the type and magnitude of stress that cause rock micro-fractures, waveforms differ in rise time, ring count, signal energy, and cross-correlation coefficients. Utilizing the similarity coefficient effectively prevents misidentifying precursor neighboring events originating from different micro-fracture sources within the sequence. Although some waveforms in certain acquisition channels exhibit deformation and signal attenuation compared to the template waveform, this has minimal impact on template-based event identification. This demonstrates that the proposed method can still accurately identify waveforms within each channel despite a certain degree of propagation attenuation.

(4) Arrival picking

**Figure 22:**
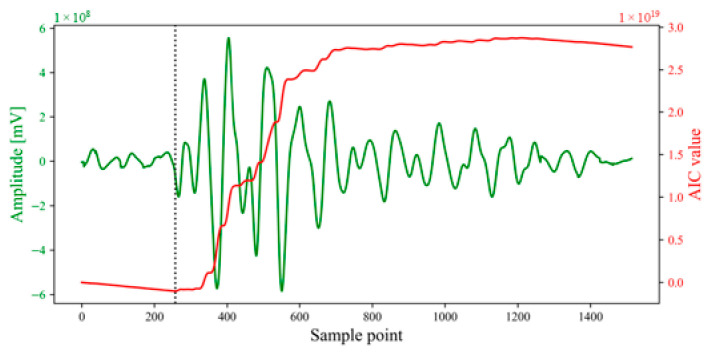
Demonstration of arrival picking for double-peak multi-waveforms. The dotted line indicates the sample point corresponding to the arrival determined based on the AIC value.

**Figure 23:**
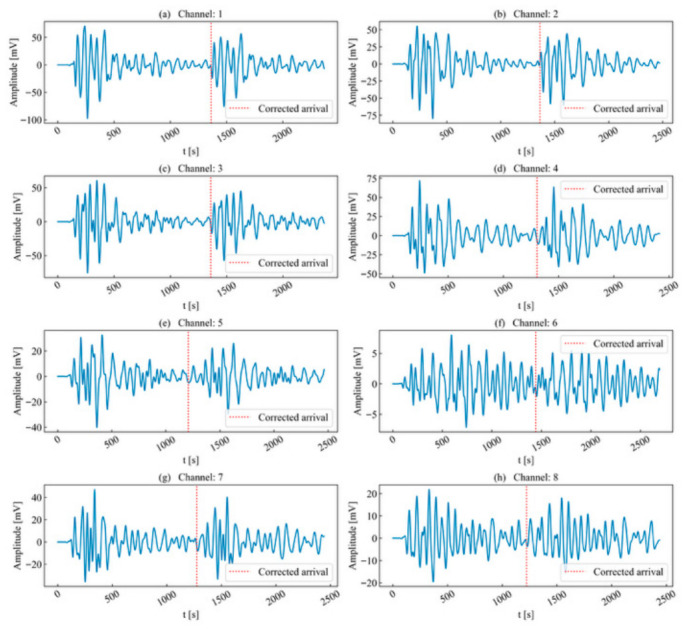
Arrival correction of double-peak multi-waveforms.

The arrival within the most similar window for each channel was selected as the reference point to calculate the corresponding time-difference correction values $t_{i}$, as presented in Table 4. Based on these correction values, each unit time series was shifted. The minimum and maximum values of the left and right boundaries of each window were taken to define the temporal superposition window, with its length set to 1514 μs. Arrival-time picking was performed within the superposition window to obtain the arrival AIC = 257 μs, as illustrated in Figure 22. Subsequently, the arrival of the superposition window was back-calculated using the time-difference correction value $t_{i}$ to derive the actual arrival times within each unit time series. As shown in Figure 23, the determined arrival times for each channel were generally near the seismic origin.

In summary, compared to the traditional threshold-based timing parameter method, the proposed method not only effectively separates double-peak multi-waveforms, thereby resolving the difficulty of separating short-interval waveforms, but also achieves arrival picking in high-SNR environments through time-difference correction and window superposition. This is of significant importance for subsequent research on waveform localization.

## 6. Conclusions

This paper proposes an AE event identification technique for rocks based on waveform energy envelopes and characteristic parameters, aiming to address the identification of dense acoustic emission events during rock fracturing processes. The method segments signal waveforms in time series using waveform energy envelopes, combined with characteristic parameters such as ring count, rise time, and signal energy, thereby achieving effective identification of signals throughout the evolution of rock fracture. Additionally, to improve the accuracy of arrival picking, a time-difference correction method is employed to effectively eliminate the temporal deviations caused by signal propagation differences across channels.

Experimental results demonstrate that the proposed method exhibits identification capability. Furthermore, the introduction of the time difference correction method significantly improves the accuracy of multi-channel event arrival time extraction, demonstrating excellent robustness, especially in multi-channel synchronous detection. The present results indicate that the proposed method is effective for representative single-peak and multi-peak waveform cases under laboratory rock-failure test conditions. It provides a methodological basis for AE signal identification and arrival-time extraction in rock-failure monitoring. However, its applicability to more complex field-scale environments, stronger noise conditions, and broader early-warning scenarios still requires further validation. Future research will further optimize the method’s performance and adaptability, exploring its application potential in larger-scale and more complex environments to provide more precise technical assurance for safety monitoring and stability assessment in geotechnical engineering.

## Figures and Tables

**Table 1:** The SNR and signal energy of single-peak single-waveforms.

Channel	1	2	3	4	5	6	7	8
SNR	20.64	20.82	20.74	14.87	16.95	11.26	15.97	14.05
Signal energy ($10^{6}$)	3.10	3.26	3.20	2.02	2.28	1.28	1.82	1.88

**Table 2:** Time-difference correction value of single-peak single-waveforms.

Channel	1	2	3	4	5	6	7	8
Time-difference correction value ($10^{-7}s$)	−120	−98	−106	−94	−70	−92	18	82
CV	7.5%	6.125%	6.625%	5.875%	4.375%	5.75%	1.125%	5.125

**Table 3:** The SNR and signal energy of double-peak multi-waveforms.

Channel	1	2	3	4	5	6	7	8
SNR	33.96	32.83	32.07	32.19	26.94	16.35	28.43	33.96
Signal energy ($10^{6}$)	3.99	3.54	3.27	3.61	2.28	0.83	2.86	3.99

**Table 4:** Time-difference correction value of double-peak multi-waveforms.

Channel	1	2	3	4	5	6	7	8
Time-difference correction value ($10^{-7}s$)	−2	−2	−6	−61	−157	76	−91	−2
CV	0.14%	0.14%	0.42%	4.35%	9.81%	5.42%	6.5%	0.14%

## Data Availability

The original contributions presented in this study are included in the article. Further inquiries can be directed to the corresponding author.
